# Notes on Preparations for the Sick

**Published:** 1906-03

**Authors:** 


### Notes on Preparations for the Sick.

**Steriloid Milk (Powder).**—British Steriloid Milk Co. Ltd., 48 Dawson Street, Dublin.—This is a yellow flaky powder, prepared from whole milk and guaranteed to contain 27 per cent. butter fat in the dried state. It is said to be sterilised, and to keep well for fourteen days. It is directed to be made with boiling water into a paste, and then reduced to the specific gravity of new milk. We have found the proportions recommended, viz. one ounce of the powder to eight fluid ounces of milk, produces a liquid of too low a specific gravity. About six ounces of water to the ounce gives a specific gravity of 1030–1035 at 15.5° C. We prepared samples of the milk, using strict antiseptic precautions, and found that at incubator temperature it kept about three days, and at room temperature (50°–55° F.) about five days. If, however, the product was boiled for fifteen minutes, it was perfectly sweet at the end of fifteen days. We also prepared samples of various specific gravity and estimated the fat. The specific gravity was taken at 15.5° C. The results showed: Sp. gr. 1035, fat 2.2 per cent. sp. gr. 1030, fat 2 per cent.; sp. gr. 1025, fat 1·4 per cent.

The flavour of this preparation closely resembles that of sterilised milk. When carefully prepared it yields a homogeneous fluid, but on standing for some hours there is a tendency for a deposit to separate out. The clot formed by rennet is very slight and flocculent.

---

**Lysoform.**—BRITISH LYSOFORM CO., London.—This substance, which has been shown to be a powerful antiseptic and germicide, should be a valuable addition to practical therapeutics. Its great advantage lies in the fact that it has little toxic power, and may thus be used with safety when substances like perchloride of mercury might lead to unpleasant results. It is also non-irritant to the hands, and as it is a powerful deodorant, is remarkably suited to the needs of the out-patient physician or surgeon or the pathologist in the form of a lotion—2 or 3 per cent. being quite strong enough for these purposes. As an ordinary surgical antiseptic stronger solutions may be used. A 5 per cent. solution destroys pyogenic cocci, and is sufficiently powerful as a lotion for wounds and antiseptic compresses.

---

**The Vapour Method of Anæsthesia.**—Dr. JAMES T. GWATHMEY, of New York, sends us a notice of a new apparatus which he has invented for the vapour method of anæsthetising, and he claims for his instrument the following "unique features":—That chloroform or ether can be given singly or combined in any desired proportion; the ability to increase or decrease the air or oxygen without at the same time increasing or decreasing the anæsthetic; the mask is an anatomically correct fitting facepiece, the rim of which is hollow and perforated around the inner margin to allow the vapour to escape, otherwise identical with a folding Esmarch mask. There is an arrangement which allows of a few drops of chloroform being added if necessary during the induction stage, as, for instance, in the case of a vigorous alcoholic patient. The inhaler gives a maximum chloroform vapour of 2 per cent. and a minimum of 0.1 per cent.

---

**Robinson's Prepared Barley.**—KEEN, ROBINSON & Co., London. —This is so well known to the medical profession, and has been in use for so many years, that its utility has been thoroughly established by actual experiment. It may be used in many ways, but is especially useful for the preparation of barley water, which in the case of young children is very beneficial in preventing the clotting of milk in the stomach, and with older children and adults is also of some value as a food, and can generally be readily digested.

An examination shows that it consists largely of carbohydrate in the form of barley starch, hence when required for feeding purposes it should be used in conjunction with milk, when a highly nutritious product results.

---

**Robinson's Groats.**—Like the preceding preparation, "Robinson's Groats" has for many years been a household name. Whereas "Prepared Barley" is essentially an adjunct to food, this preparation possesses more of the characteristics of a true food, in that it contains a good proportion of proteid matter. Judging from a microscopic examination, oatmeal figures prominently in its composition, the starch grains having a very characteristic appearance. It yields a very appetising gruel, which has many valuable properties, and which in most cases is readily digested, even by delicate persons. A preparation which has stood the test of time can but receive our approbation.

---

**Mustard Bran; Mustard Leaves; Mustard Oil.**—J. & J. COLMAN, 108 Cannon Street, London.—The latter preparation has been before the public for a considerable number of years, and has gained a reputation as one of the best stimulating liniments known to those concerned in such matters.

Its stimulating effect is largely due to the presence of essential oil of mustard, a pungent body known chemically as "allyl isothiocyanate." Whilst acting as a somewhat vigorous stimulant, it is not liable to cause any unpleasant effects, and can be used with safety even in the case of young children.

---

**Brand's Nutrient Powder** (Deuce's Patent).—BRAND & Co. LTD., Mayfair, W.—The above preparation is recommended by the makers as a useful addition to the diet of invalids and others requiring specially nourishing food. The quantity for administration is one teaspoonful or more, mixed with milk, beef tea, &c. A chemical examination shows that it consists chiefly of proteid, but it is not very soluble in cold water.

An experiment was made by mixing a little of the powder with water and a trace of hydrochloric acid and pepsin, the temperature being about that of the body, or a trifle higher. We consider that experiments "in vitreo" with such bodies are rather severe, but the result was satisfactory. Solution soon began to take place, and after a time the resulting product was found to be rich in peptone. Judging from these results, it should be readily digested and assimilated by the alimentary system.

---

**Emulsion of Cod Liver Oil.**—W. Pitchford & Son, Bristol.—This is a specially prepared emulsion, containing 50 per cent. of the finest Norwegian oil, the emulsifying agent being eggs. It does not undergo separation on standing, a common fault with many of the usual preparations of this type. The taste of the oil is well disguised, elixir of saccharin and almond being among the flavouring agents. It can be recommended where a palatable and reliable preparation of cod liver oil is required.

---

**Pleated Compressed Sanitary Towels** ("Tabloid" Brand).—Burroughs, Wellcome & Co., London.—These possess several points of superiority over ordinary commercial sanitary towels, and are in every way the most convenient and satisfactory. They are made of materials of exceptional quality, specially prepared for the required purpose, their highly absorbent properties being particularly noteworthy. The delicate texture of the outer covering ensures absolute freedom from the slightest sense of discomfort in use. After being highly compressed each is enclosed in an efficient protective covering, perfect cleanliness being thus secured. The extremely small space which they occupy renders them particularly convenient when travelling. Four sizes are issued—Nos. 1, 2, 3 and 4, of which No. 4 is the largest. Each size is issued in packages of one dozen.

---

**Soloids (Eucaine Lactate).**—Burroughs, Wellcome & Co., London.—Of the salts of eucaine the lactate is the most generally useful. In anæsthetic power it is equal to cocaine salts, but is non-toxic, non-irritating, and does not constrict the blood-vessels or dilate the pupil. It is extremely soluble, 20 per cent. solutions being readily made with cold water. The stability of ts solutions is such that they may be sterilised by boiling without undergoing decomposition.

Two per cent. solutions are used for ophthalmic purposes, and for laryngoscopy and rhinoscopy; 4 to 8 per cent. for the nose previous to the use of the galvano-cautery; 6 to 8 per cent. for removal of tonsils; and 2 to 4 per cent. for catheterisation and for endoscopic examinations. In dental work 2 per cent. solutions are commonly used. In general surgery, eucaine is used as a local anæsthetic by hypodermic injection of 4 per cent. solutions. Though the strengths of solution here indicated are those commonly employed, yet on occasion 20 per cent., and even much stronger, may be desirable.

The following preparations are issued: "Tabloid" Hypodermic Eucaine Lactate, gr. $\frac{1}{3}$ and gr. 1; "Soloid" Eucaine Lactate, gr. 1 and gr. 5.

